# 1-Ethyl-4-hydr­oxy-9-aza­tricyclo­[7.4.1.0^2,7^]tetra­deca-2,4,6-trien-8-one

**DOI:** 10.1107/S160053680903918X

**Published:** 2009-11-07

**Authors:** Wei Zheng, Qiong Xie, Fang Li, Zhui-Bai Qiu

**Affiliations:** a Department of Medicinal Chemistry, School of Pharmacy, Fudan University, 826 Zhangheng Road, Shanghai 201203, People’s Republic of China; b NPFPC Key Laboratory of Contraceptives and Devices, Shanghai Institute of Planned Parenthood Research, 2140 Xietu Road, Shanghai 200032, People’s Republic of China

## Abstract

In the mol­ecule of the title compound, C_15_H_19_NO_2_, the six-membered dihydro­pyridinone ring assumes a screw-boat conformation. In the crystal structure, mol­ecules are linked *via* O—H⋯O hydrogen bonding between hydr­oxy and carbonyl groups, forming supra­molecular chains along the *a* axis.

## Related literature

For the synthesis and bioactivity of novel bis-(−)-*nor*-meptazinols, see Xie *et al.* (2008 ▶).
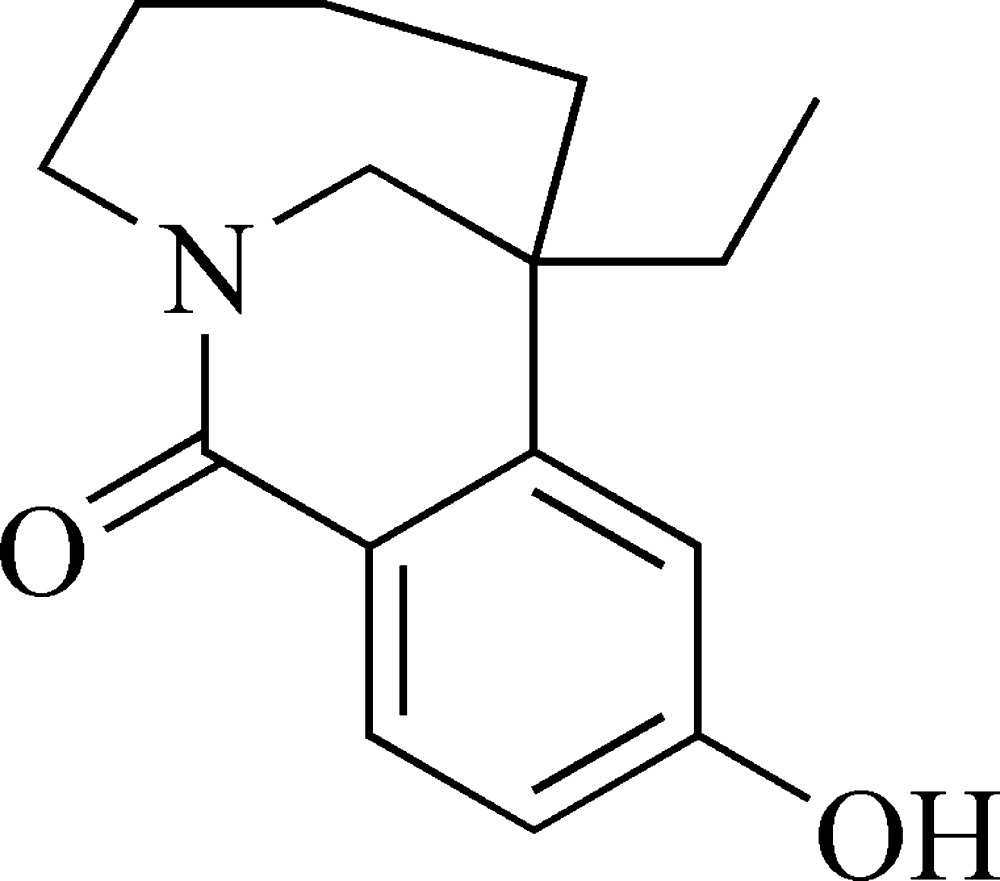


## Experimental

### 

#### Crystal data

C_15_H_19_NO_2_
*M* *_r_* = 245.31
Orthorhombic, 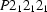

*a* = 8.298 (1) Å
*b* = 9.9817 (12) Å
*c* = 14.7324 (18) Å
*V* = 1220.3 (3) Å^3^
*Z* = 4
Mo *K*α radiation
μ = 0.09 mm^−1^
*T* = 293 K
0.37 × 0.23 × 0.22 mm

#### Data collection

Bruker SMART APEX CCD area-detector diffractometer
Absorption correction: none
6713 measured reflections
1397 independent reflections
1280 reflections with *I* > 2σ(*I*)
*R*_int_ = 0.054

#### Refinement

*R*[*F* ^2^ > 2σ(*F* ^2^)] = 0.038
*wR*(*F* ^2^) = 0.091
*S* = 1.06
1397 reflections
165 parameters
H-atom parameters constrained
Δρ_max_ = 0.16 e Å^−3^
Δρ_min_ = −0.20 e Å^−3^

### 

Data collection: *SMART* (Bruker, 2000 ▶); cell refinement: *SAINT* (Bruker, 2000 ▶); data reduction: *SAINT*; program(s) used to solve structure: *SHELXTL* (Sheldrick, 2008 ▶); program(s) used to refine structure: *SHELXTL*; molecular graphics: *SHELXTL*; software used to prepare material for publication: *SHELXTL*.

## Supplementary Material

Crystal structure: contains datablocks global, I. DOI: 10.1107/S160053680903918X/xu2615sup1.cif

Structure factors: contains datablocks I. DOI: 10.1107/S160053680903918X/xu2615Isup2.hkl

Additional supplementary materials:  crystallographic information; 3D view; checkCIF report

## Figures and Tables

**Table 1:** Hydrogen-bond geometry (Å, °)

*D*—H⋯*A*	*D*—H	H⋯*A*	*D*⋯*A*	*D*—H⋯*A*
O2—H2⋯O1^i^	0.82	1.92	2.710 (2)	162

Symmetry code: (i) 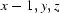.

**Fig. 1.**
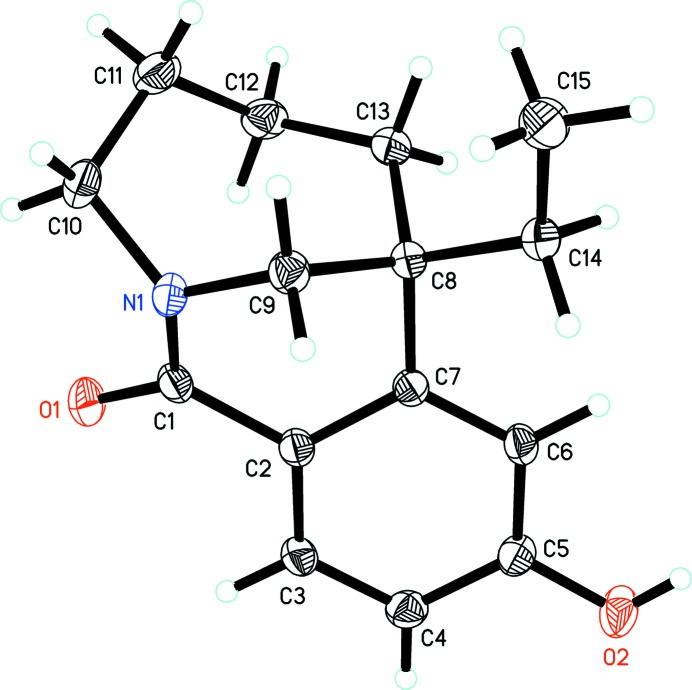
A view of the structure of title compound, showing the atomic numbering scheme. Displacement ellipsoids are drawn at the 50% probability level.

**Fig. 2.**
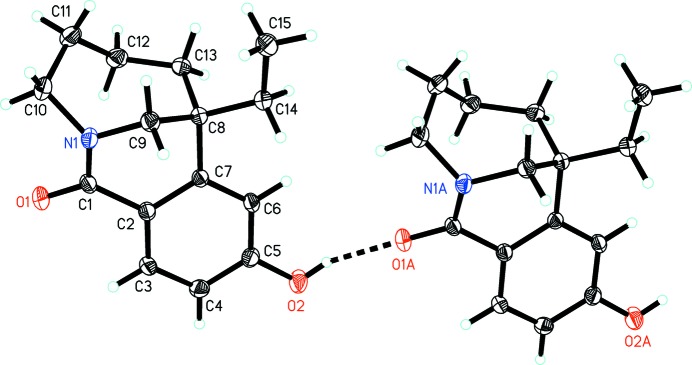
Intermolecular hydrogen bond is shown as a dashed line.

**Fig. 3.**
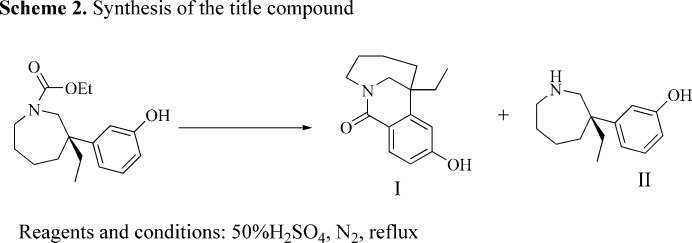
The formation of the title compound.

## supplementary crystallographic information

### Comment

In previous study, our group has reported the synthesis and characterization of novel bis-(-)-*nor*-meptazinols that inhibit both acetylcholinesterase and butyrylcholinesterase as well as retarding Aβ aggregation (Xie *et al.*, 2008). The title compound (I), was produced serendipitously whilst preparating of (-)-Nor-meptazinol (II), which is the intermediate of bis-(-)-*nor*-meptazinols. The molecular structure of (I) and the atom-numbering scheme are shown in Fig. 1. The six-membered dihydropyridinone ring assumes a screw-boat conformation. The crystal structure is stabilized by intermolecular O—H···O hydrogen bonding (Table 1 and Fig. 2).

### Experimental

A white powder (I) was obtained as a by-product of the reaction between (-)-*N*-carboethoxy-nor-meptazinol and 50% H_2_SO_4_ (Fig. 3). Single crystals suitable for crystallographic analysis were obtained by slow evaporation of an methanol solution. [α]_D_ = -60.8° (c 0.332, MeOH).

### Refinement

All H atoms were positioned geometrically and refined as riding (C—H = 0.93–0.97 Å, O—H = 0.82 Å), with U_iso_(H) = 1.2U_eq_(C) and 1.5U_eq_(O). In the absence of significant anomalous scattering, Friedel opposites were merged and the absolute structure is not determined.

### Crystal data

**Table:**

C_15_H_19_NO_2_	*F*(000) = 528
*M**_r_* = 245.31	*D*_x_ = 1.335 Mg m^−^^3^
Orthorhombic, *P*2_1_2_1_2_1_	Mo *K*α radiation, λ = 0.71073 Å
Hall symbol: P 2ac 2ab	Cell parameters from 2536 reflections
*a* = 8.298 (1) Å	θ = 2.5–26.4°
*b* = 9.9817 (12) Å	µ = 0.09 mm^−^^1^
*c* = 14.7324 (18) Å	*T* = 293 K
*V* = 1220.3 (3) Å^3^	Prismatic, colourless
*Z* = 4	0.37 × 0.23 × 0.22 mm

### Data collection

**Table:**

Bruker SMART APEX CCD area-detector diffractometer	1280 reflections with *I* > 2σ(*I*)
Radiation source: fine-focus sealed tube	*R*_int_ = 0.054
graphite	θ_max_ = 26.0°, θ_min_ = 2.5°
φ and ω scans	*h* = −10→10
6713 measured reflections	*k* = −12→12
1397 independent reflections	*l* = −11→18

### Refinement

**Table:**

Refinement on *F*^2^	Primary atom site location: structure-invariant direct methods
Least-squares matrix: full	Secondary atom site location: difference Fourier map
*R*[*F*^2^ > 2σ(*F*^2^)] = 0.038	Hydrogen site location: inferred from neighbouring sites
*wR*(*F*^2^) = 0.091	H-atom parameters constrained
*S* = 1.06	*w* = 1/[σ^2^(*F*_o_^2^) + (0.0557*P*)^2^] where *P* = (*F*_o_^2^ + 2*F*_c_^2^)/3
1397 reflections	(Δ/σ)_max_ < 0.001
165 parameters	Δρ_max_ = 0.16 e Å^−^^3^
0 restraints	Δρ_min_ = −0.20 e Å^−^^3^

### Special details

**Table:**

Geometry. All e.s.d.'s (except the e.s.d. in the dihedral angle between two l.s. planes) are estimated using the full covariance matrix. The cell e.s.d.'s are taken into account individually in the estimation of e.s.d.'s in distances, angles and torsion angles; correlations between e.s.d.'s in cell parameters are only used when they are defined by crystal symmetry. An approximate (isotropic) treatment of cell e.s.d.'s is used for estimating e.s.d.'s involving l.s. planes.
Refinement. Refinement of *F*^2^ against ALL reflections. The weighted *R*-factor *wR* and goodness of fit *S* are based on *F*^2^, conventional *R*-factors *R* are based on *F*, with *F* set to zero for negative *F*^2^. The threshold expression of *F*^2^ > σ(*F*^2^) is used only for calculating *R*-factors(gt) *etc*. and is not relevant to the choice of reflections for refinement. *R*-factors based on *F*^2^ are statistically about twice as large as those based on *F*, and *R*- factors based on ALL data will be even larger.

### Fractional atomic coordinates and isotropic or equivalent isotropic displacement parameters (Å^2^)

**Table:**

	*x*	*y*	*z*	*U*_iso_*/*U*_eq_	
N1	1.0502 (2)	0.76044 (17)	0.90237 (11)	0.0315 (4)	
O1	1.16080 (18)	0.96697 (17)	0.91934 (11)	0.0429 (4)	
O2	0.4251 (2)	1.11984 (16)	0.94981 (13)	0.0507 (5)	
H2	0.3557	1.0686	0.9307	0.076*	
C1	1.0402 (2)	0.8931 (2)	0.91820 (13)	0.0301 (5)	
C2	0.8763 (2)	0.9473 (2)	0.93043 (13)	0.0285 (5)	
C3	0.8531 (3)	1.0689 (2)	0.97449 (13)	0.0328 (5)	
H3	0.9410	1.1126	1.0001	0.039*	
C4	0.7027 (3)	1.1258 (2)	0.98083 (14)	0.0357 (5)	
H4	0.6883	1.2061	1.0118	0.043*	
C5	0.5729 (3)	1.0620 (2)	0.94053 (15)	0.0336 (5)	
C6	0.5946 (3)	0.9430 (2)	0.89339 (14)	0.0310 (5)	
H6	0.5072	0.9031	0.8646	0.037*	
C7	0.7454 (2)	0.8827 (2)	0.88863 (12)	0.0262 (4)	
C8	0.7732 (2)	0.7468 (2)	0.84288 (13)	0.0279 (4)	
C9	0.9025 (3)	0.67983 (19)	0.90089 (14)	0.0312 (5)	
H9A	0.8627	0.6687	0.9623	0.037*	
H9B	0.9263	0.5917	0.8766	0.037*	
C10	1.1831 (3)	0.7095 (2)	0.84858 (15)	0.0416 (6)	
H10A	1.2152	0.6222	0.8711	0.050*	
H10B	1.2747	0.7694	0.8537	0.050*	
C11	1.1318 (3)	0.6983 (2)	0.74892 (15)	0.0431 (6)	
H11A	1.2261	0.7101	0.7108	0.052*	
H11B	1.0905	0.6087	0.7382	0.052*	
C12	1.0037 (3)	0.7998 (2)	0.72030 (14)	0.0386 (6)	
H12A	1.0121	0.8133	0.6553	0.046*	
H12B	1.0277	0.8847	0.7494	0.046*	
C13	0.8296 (3)	0.7620 (2)	0.74266 (13)	0.0336 (5)	
H13A	0.8074	0.6777	0.7124	0.040*	
H13B	0.7609	0.8288	0.7146	0.040*	
C14	0.6173 (3)	0.6630 (2)	0.84401 (15)	0.0351 (5)	
H14A	0.5664	0.6741	0.9028	0.042*	
H14B	0.5444	0.6993	0.7988	0.042*	
C15	0.6367 (3)	0.5130 (2)	0.82599 (18)	0.0477 (6)	
H15A	0.6932	0.5000	0.7698	0.072*	
H15B	0.5323	0.4719	0.8223	0.072*	
H15C	0.6968	0.4729	0.8746	0.072*	

### Atomic displacement parameters (Å^2^)

**Table:**

	*U*^11^	*U*^22^	*U*^33^	*U*^12^	*U*^13^	*U*^23^
N1	0.0219 (9)	0.0378 (9)	0.0348 (9)	0.0014 (8)	−0.0012 (7)	−0.0003 (8)
O1	0.0258 (8)	0.0495 (9)	0.0534 (9)	−0.0093 (7)	−0.0018 (7)	−0.0064 (8)
O2	0.0298 (9)	0.0380 (9)	0.0842 (13)	0.0030 (8)	0.0012 (9)	−0.0190 (9)
C1	0.0256 (11)	0.0373 (11)	0.0273 (10)	−0.0054 (9)	−0.0036 (9)	−0.0018 (9)
C2	0.0254 (11)	0.0343 (11)	0.0259 (9)	−0.0029 (9)	−0.0007 (8)	0.0007 (8)
C3	0.0286 (11)	0.0367 (11)	0.0330 (10)	−0.0075 (10)	−0.0013 (9)	−0.0048 (9)
C4	0.0375 (13)	0.0308 (11)	0.0387 (11)	−0.0019 (10)	0.0035 (10)	−0.0071 (9)
C5	0.0287 (11)	0.0301 (10)	0.0421 (12)	−0.0003 (10)	0.0040 (9)	−0.0020 (9)
C6	0.0243 (11)	0.0315 (10)	0.0371 (11)	−0.0044 (9)	−0.0026 (9)	−0.0034 (9)
C7	0.0247 (11)	0.0311 (10)	0.0229 (9)	−0.0037 (9)	0.0012 (8)	0.0012 (8)
C8	0.0239 (11)	0.0322 (10)	0.0276 (9)	−0.0013 (9)	−0.0003 (8)	−0.0035 (8)
C9	0.0294 (11)	0.0303 (10)	0.0338 (11)	−0.0018 (9)	−0.0003 (9)	−0.0007 (9)
C10	0.0241 (12)	0.0475 (13)	0.0532 (14)	0.0042 (10)	0.0015 (10)	−0.0066 (11)
C11	0.0361 (14)	0.0462 (12)	0.0470 (13)	0.0007 (11)	0.0124 (11)	−0.0097 (11)
C12	0.0425 (14)	0.0433 (12)	0.0302 (10)	−0.0039 (12)	0.0068 (10)	−0.0011 (9)
C13	0.0328 (12)	0.0406 (11)	0.0274 (10)	−0.0005 (10)	−0.0011 (9)	−0.0042 (9)
C14	0.0271 (12)	0.0391 (11)	0.0390 (11)	−0.0039 (10)	0.0015 (10)	−0.0078 (9)
C15	0.0402 (15)	0.0392 (12)	0.0637 (15)	−0.0090 (11)	0.0056 (12)	−0.0131 (11)

### Geometric parameters (Å, °)

**Table:**

N1—C1	1.347 (3)	C9—H9A	0.9700
N1—C10	1.451 (3)	C9—H9B	0.9700
N1—C9	1.466 (3)	C10—C11	1.533 (3)
O1—C1	1.243 (2)	C10—H10A	0.9700
O2—C5	1.362 (3)	C10—H10B	0.9700
O2—H2	0.8200	C11—C12	1.528 (3)
C1—C2	1.475 (3)	C11—H11A	0.9700
C2—C3	1.390 (3)	C11—H11B	0.9700
C2—C7	1.406 (3)	C12—C13	1.529 (3)
C3—C4	1.375 (3)	C12—H12A	0.9700
C3—H3	0.9300	C12—H12B	0.9700
C4—C5	1.385 (3)	C13—H13A	0.9700
C4—H4	0.9300	C13—H13B	0.9700
C5—C6	1.387 (3)	C14—C15	1.529 (3)
C6—C7	1.391 (3)	C14—H14A	0.9700
C6—H6	0.9300	C14—H14B	0.9700
C7—C8	1.532 (3)	C15—H15A	0.9600
C8—C9	1.526 (3)	C15—H15B	0.9600
C8—C14	1.541 (3)	C15—H15C	0.9600
C8—C13	1.556 (3)		
			
C1—N1—C10	119.04 (19)	N1—C10—C11	109.72 (18)
C1—N1—C9	119.42 (17)	N1—C10—H10A	109.7
C10—N1—C9	115.80 (16)	C11—C10—H10A	109.7
C5—O2—H2	109.5	N1—C10—H10B	109.7
O1—C1—N1	122.43 (19)	C11—C10—H10B	109.7
O1—C1—C2	121.54 (18)	H10A—C10—H10B	108.2
N1—C1—C2	115.98 (18)	C12—C11—C10	114.14 (17)
C3—C2—C7	119.91 (19)	C12—C11—H11A	108.7
C3—C2—C1	120.34 (18)	C10—C11—H11A	108.7
C7—C2—C1	119.38 (17)	C12—C11—H11B	108.7
C4—C3—C2	121.2 (2)	C10—C11—H11B	108.7
C4—C3—H3	119.4	H11A—C11—H11B	107.6
C2—C3—H3	119.4	C11—C12—C13	115.73 (18)
C3—C4—C5	119.17 (18)	C11—C12—H12A	108.3
C3—C4—H4	120.4	C13—C12—H12A	108.3
C5—C4—H4	120.4	C11—C12—H12B	108.3
O2—C5—C4	117.53 (18)	C13—C12—H12B	108.3
O2—C5—C6	122.03 (19)	H12A—C12—H12B	107.4
C4—C5—C6	120.4 (2)	C12—C13—C8	120.83 (17)
C5—C6—C7	120.88 (19)	C12—C13—H13A	107.1
C5—C6—H6	119.6	C8—C13—H13A	107.1
C7—C6—H6	119.6	C12—C13—H13B	107.1
C6—C7—C2	118.32 (17)	C8—C13—H13B	107.1
C6—C7—C8	122.77 (18)	H13A—C13—H13B	106.8
C2—C7—C8	118.88 (18)	C15—C14—C8	116.17 (18)
C9—C8—C7	104.31 (15)	C15—C14—H14A	108.2
C9—C8—C14	110.29 (16)	C8—C14—H14A	108.2
C7—C8—C14	110.45 (16)	C15—C14—H14B	108.2
C9—C8—C13	111.28 (17)	C8—C14—H14B	108.2
C7—C8—C13	112.08 (17)	H14A—C14—H14B	107.4
C14—C8—C13	108.41 (16)	C14—C15—H15A	109.5
N1—C9—C8	110.83 (15)	C14—C15—H15B	109.5
N1—C9—H9A	109.5	H15A—C15—H15B	109.5
C8—C9—H9A	109.5	C14—C15—H15C	109.5
N1—C9—H9B	109.5	H15A—C15—H15C	109.5
C8—C9—H9B	109.5	H15B—C15—H15C	109.5
H9A—C9—H9B	108.1		
			
C10—N1—C1—O1	−27.0 (3)	C2—C7—C8—C9	33.5 (2)
C9—N1—C1—O1	−179.22 (17)	C6—C7—C8—C14	−25.7 (2)
C10—N1—C1—C2	150.53 (17)	C2—C7—C8—C14	152.04 (18)
C9—N1—C1—C2	−1.7 (3)	C6—C7—C8—C13	95.3 (2)
O1—C1—C2—C3	−23.0 (3)	C2—C7—C8—C13	−87.0 (2)
N1—C1—C2—C3	159.45 (18)	C1—N1—C9—C8	47.4 (2)
O1—C1—C2—C7	150.1 (2)	C10—N1—C9—C8	−105.69 (19)
N1—C1—C2—C7	−27.5 (3)	C7—C8—C9—N1	−59.9 (2)
C7—C2—C3—C4	2.2 (3)	C14—C8—C9—N1	−178.47 (15)
C1—C2—C3—C4	175.27 (19)	C13—C8—C9—N1	61.2 (2)
C2—C3—C4—C5	−1.6 (3)	C1—N1—C10—C11	−94.0 (2)
C3—C4—C5—O2	178.7 (2)	C9—N1—C10—C11	59.2 (2)
C3—C4—C5—C6	−0.8 (3)	N1—C10—C11—C12	29.3 (3)
O2—C5—C6—C7	−176.9 (2)	C10—C11—C12—C13	−82.8 (2)
C4—C5—C6—C7	2.6 (3)	C11—C12—C13—C8	63.9 (3)
C5—C6—C7—C2	−1.9 (3)	C9—C8—C13—C12	−39.0 (3)
C5—C6—C7—C8	175.79 (18)	C7—C8—C13—C12	77.3 (2)
C3—C2—C7—C6	−0.5 (3)	C14—C8—C13—C12	−160.48 (18)
C1—C2—C7—C6	−173.55 (19)	C9—C8—C14—C15	−48.4 (3)
C3—C2—C7—C8	−178.25 (17)	C7—C8—C14—C15	−163.21 (19)
C1—C2—C7—C8	8.6 (3)	C13—C8—C14—C15	73.6 (2)
C6—C7—C8—C9	−144.15 (19)		

### Hydrogen-bond geometry (Å, °)

**Table:**

*D*—H···*A*	*D*—H	H···*A*	*D*···*A*	*D*—H···*A*
O2—H2···O1^i^	0.82	1.92	2.710 (2)	162

Symmetry codes: (i) *x*−1, *y*, *z*.
